# Challenges and solutions for instituting an efficient maintenance program for laboratory equipment in Central Asian, and developing world, countries

**DOI:** 10.1186/s12889-019-6782-5

**Published:** 2019-05-10

**Authors:** Riza Ikranbegiin, George Schmid, David Hoos, Andrew Young, Phyllis Della-Latta, Paul Spearman, Artur Ramos, Bereket Alemayehu, Begaiym Achmetova, Gulzhan Nauryzova, Adilya Albetkova

**Affiliations:** 1 World Health Organization, Regional Office for Europe, Copenhagen, Denmark; 2 Centers for Disease Control and Prevention, Atlanta, GA USA; 3 ICAP Global Health Action, Columbia University, Mailman School of Public Health, New York, NY USA; 4 Laboratory of Grady Health System, Grady Memorial Hospital, Atlanta, GA USA; 5 Clinical Microbiology Service, Columbia University Medical Center NewYork-Presbyterian Hospital, New York, NY USA; 6 Department of Pediatrics, Emory University, Children’s Healthcare of Atlanta, Atlanta, GA USA; 7 ICAP Global Health Action, Columbia University, Mailman School of Public Health, New York, USA; 8 Clinical Diagnostic Laboratory of the Almaty AIDS Center, 2 Basenov Str., Almaty, Kazakhstan; 9 ICAP Global Health Action, Columbia University, Mailman School of Public Health, Bishkek, Kyrgyzstan

## Abstract

We review the current state of quality assurance in laboratories of the five Central Asia Republics (CARs), focusing on laboratory equipment, and compare quality assurance approaches with CLSI standards. The laboratories of the CARs faced exceptional challenges including highly-structured laboratory systems that retain centralized and outmoded Soviet-era approaches to quality assurance, considerably jeopardizing the validity of laboratory tests. The relative isolation of the CARs, based on geography and almost exclusive use of the Russian language, further hamper change. CARs must make high-level government decisions to widely implement quality assurance programs within their laboratory systems, within which approaches to the management of laboratory equipment will be a prominent part.

## Background

At the crossroads between Europe and Asia, the Central Asia Republics (CARs) have received increased (but insufficient) attention from the international community due to the political and economic significance of the region. These countries – Kazakhstan, Kyrgyzstan, Tajikistan, Turkmenistan and Uzbekistan – have faced enormous challenges in establishing and stabilizing their institutions since attaining independence in 1991, following the dissolution of the Soviet Union. Challenges involve governance - as authority has transitioned from highly standardized Soviet bureaucracies to more independent political structures at regional, national, provincial and local levels – and demographic. Although overall population density is low, many men have left - to work in Russia or elsewhere, leaving villages without men; poverty by national standards is widespread in all countries [1], particularly in rural areas; health care systems are poor [2–6]; and a growing percentage of the population is moving to cities to escape poverty and adopting Western lifestyles and diets, creating new health challenges.

Health care systems are changing but the traditional laboratory structures have operated from a centralized, national level, with oversight of service quality generally conducted by national ministries of health. In these dynamic societies, diagnostic services for patients will increasingly need to occur in local, non-specialized laboratories based in hospitals or clinics. The delivery of laboratory services to neglected rural populations requires additional decentralization. Based on these trends, quality management of clinical laboratories – including the management of equipment – will require attention at the local level, which is the model used for laboratories in developed countries and one espoused for health care systems by the World Health Organization European Office as it assists countries in the former Soviet Union [7].

The appropriate diagnosis and then management of illness requires valid test results. Valid laboratory results are also necessary for rapid detection and control of outbreaks of infectious diseases and other public health threats at their source and therefore essential for global health security but valid test results can be elusive. It is often thought that ex-Soviet health care systems are near-equivalents of Western systems…they are not. It is common in the CARs, for example, for people in cities with financial means to “laboratory shop” by visiting multiple laboratories, comparing conflicting results in an attempt to decipher what might be correct results. Poor people do not have even this poor option. Testing exists, but quality is uncertain.

Little is written about the quality of laboratory services in Central Asia (a search using “Central Asia health laboratory quality” with Google Scholar yielded no relevant article in the first 100 articles identified). Yet, our experience and that of others shows the need for Quality Management System (QMS) implementation in our region is big [8–10] and our observations important. The combined population of the five CARs is 61 million people in a region that retains an ex-Soviet heritage and which is relatively isolated from world literature by near-exclusive use of the Russian language. The challenges found in these countries are likely to be present in other countries with a similar heritage and similar systems [11].

In this paper, we provide a comprehensive review of QMS and its application within laboratories in the CARs, and then follow this with our observations regarding how widely used international standards are within the CARs and the need for their use there.

### Quality management of clinical laboratories

International organizations have long supported disease-specific, “siloed” laboratory systems and attendant QMS. Consistent with an increased focus on integrated services, international organizations should support an approach that focuses on strengthening key cross-cutting elements, including a practical approach to QMS [12]. Accordingly, the concept of a total-laboratory QMS has been developed through the guidelines and standards of international and national organizations with recognized expertise, such as the International Organization of Standardization (ISO), the World Health Organization (WHO), the Clinical and Laboratory Standards Institute (CLSI) and the Centers for Disease Control and Prevention (CDC) (Table 1).

**Table 1:** Reference documents for laboratory quality management systems

Organization	Document	Title
ISO	15189 17025	Medical laboratories: Particular requirements for quality and competence General requirements for the competence of testing and calibration laboratories
WHO	Handbook	Laboratory Quality Management System
CLSI	GP26 CD37	Quality management system: A Model for laboratory services Quality management system: Equipment

As defined by the ISO and CLSI, a QMS represents “coordinated activities to direct and control an organization with regard to quality.” With ISO developing standards and CLSI and WHO providing and coordinating with one another guidance on implementation of procedures to meet those standards, these QMS guidelines are consistent with one another, although they vary in degree of specificity. For example, CLSI documents often contain step-by-step suggestions, which are useful in developing countries that are establishing quality management processes for the first time.

In 2005, ISO [13] released requirements for both quality management and technical operations of testing and calibration in laboratories, followed by standards specific for the medical laboratory [14]. From 2006 to 2008, WHO, CDC and CLSI collaborated in a laboratory QMS initiative leading to publication of a CLSI guideline [15]. This guideline (GP26-A4, 2011, Fourth Edition) provides a structured approach for organizing, creating, and maintaining the management infrastructure for a quality laboratory system. Based on CLSI guidelines and the ISO 15189 standard, WHO published a handbook “Laboratory Quality Management System” [16] and tool kit “Laboratory Quality Management Systems Training Toolkit” [17], to guide and monitor laboratory QMS implementation.

The CLSI guidelines are based in part on the Clinical Laboratory Improvement Amendments (CLIA), which are American Federal laws implemented in 1988 to govern clinical laboratories in the USA. However, they are designed to be applicable to international laboratories.

Recently, step-wise international approaches to quality improvement have been developed which provide for progressive and incremental quality improvement. Such tools include the WHO Laboratory Quality Stepwise Implementation (LQSI) tool [18], which translates the ISO 15189 quality standard for medical laboratories into a stepwise process to implement a QMS, and, the WHO-Afro Strengthening Laboratory Management Towards Accreditation (SLMTA) program [19], which provides a laboratory management framework and curriculum to define and teach specific, measurable job tasks for laboratory personnel on how to manage quality in a laboratory. Progress is monitored with the Stepwise Laboratory Improvement Process Towards Accreditation (SLIPTA) checklist [19] and stepwise laboratory quality improvement is recognized based on the quantitative checklist score. The ultimate goal of these stepwise programs is to prepare laboratories in developing countries to establish sustainable quality management systems that meet international accreditation standards [20]. These tools are designed to complement one another and work in harmony to build capacities from the bench level and up in a national laboratory system and from the highest levels of decision making down through the system.

The WHO quality management model organizes all laboratory activities into 12 quality system essentials (QSEs) that serve as building blocks for coordinated and interrelated activities. Each QSE must be addressed for the overall goal of laboratory quality improvement to be achieved [16]. Failure in even one of the QSEs can result in inappropriate technical procedures.

Fundamental challenges to the quality of laboratory tests in less-resourced countries are: 1) the lack of a laboratory management infrastructure and quality management training curriculum that develops the competences of laboratory managers and quality coordinators; 2) lack of access to or knowledge of current international standards; and 3) an absence of national standard operating procedures that are based on these standards. As a consequence, there are significant quality gaps in laboratories of resource-limited countries relative to international standards. For example, these laboratories often find it very difficult to hire qualified medical technologists who are trained to follow established testing algoritms and quality control protocols, specific guidelines, workplace health regulations and instrument maintenance controls. In addition, there are few resources to conduct periodic competency testing and continuing education to assure that technologists retain core knowledge of authorized procedures and remain abreast of international and national standards.

To close these quality gaps, it will be important for developing countries and donor organizations to focus on implementing tools that assist laboratories to adopt QMS models that begin to address each of the QSEs.

### Equipment management in resource-limited countries

Against this backdrop of need in countries with limited resources, the QSE that deals with equipment management and maintenance deserves special attention. Much of the laboratory equipment in developing countries, including the CARs, is donated by international aid organizations, or purchased with their funds. However, it is rare that funds are included to maintain equipment in a state necessary to produce reliable test results. In addition, there are few standardized indicators with which donor organizations can assess developing countries on how well they address equipment management and maintenance. Based upon the ISO, CLSI, and WHO guidelines [21], equipment management systems should be characterized by formal policies, processes and procedures for selection, qualification, validation, maintenance, calibration, troubleshooting, decommission and record keeping [15, 21]. These systems ensure that a laboratory selects equipment that meets its needs; maintains it in a state that produces reliable test results; and documents its processes sufficiently for internal and external oversight. This approach has become common and has been implemented successfully in highly-resourced countries during the last 20 years. In contrast, few developing countries, including the CARs, have developed these quality management systems for effective laboratory equipment management.

### Management of laboratory equipment

The following sections describes the management of laboratory equipment in the four Central Asian countries of Kazakhstan, Uzbekistan, Tajikistan and Kyrgyzstan (while differences exist in laboratory services between the individual CARs, there are enough similarities among their QMSs that they can be compared as a group) and compares them to CLSI standards [21] for: selecting appropriate equipment; performing installation qualification; and using, calibrating, and maintaining equipment according to established schedules and processes based on the international, national, and accreditation requirements*.* The CLSI guideline developed in line with ISO 15189 provides very specific guidance on equipment management.

### Quality management systems in Central Asian countries

The overall laboratory QMS, which had been centralized from Moscow during the existence of the USSR, was not maintained during the period immediately after independence. After independence, the Ministry of Health (MOH) of each CAR gained oversight of laboratory services. But, the Constitution of each CAR left the authority for coordination of equipment management, which is one of the main part of QMS, to National Institute for Standards and Metrology (NISM). It is the responsibility of Metrology (NISM) to verify the required measurement accuracy and the functioning of the measuring system at defined intervals according to manufacturer’s instructions, also ISO requirements and to certify annually if the requirement was met. However, despite these intended levels of oversight, laboratory services experienced many funding shortfalls and the loss of highly experienced laboratory staff during the formation of the independent CARs. Currently, funding sources and levels for various laboratory networks differ, with research labs funded with competitive grants from governmental and non-governmental agencies, veterinary and “public health” laboratories supported by government funding, and clinical laboratories, while principally government-funded, are in some countries finding public-private partnerships or becoming exclusively privately funded. International donor programs have helped to build government capacity, increasingly according to “Western standards,” to improve access and delivery of clinical and veterinary laboratory services. Important developmental gaps remain, and a large gap is in including the lack of equipment management in laboratory services and the regulation of laboratories in the private sector.

### Laboratory equipment management (EM) in CARs

CLSI document QMS13-A [21] provides recommendations for criteria and methods used in all operations that occur during the typical lifecycle of laboratory equipment, including selection, identification, validation, reverification, testing, and decommission. The guideline describes each of these operations and includes many sample forms and templates for use in documenting all aspects of the equipment life-cycle.

In the CLSI QMS13-A [21] guideline, laboratory equipment can be classified into two major categories: *general laboratory equipment* and *laboratory instrumentation.* General laboratory equipment is that which can be used in various laboratory settings or methods, while laboratory instrumentation is used to produce measurements in a specific examination/analytical system or method (Table 2). The distinction is useful. General laboratory equipment is often used for many purposes, and does not need frequent calibration or careful quality control. Laboratory instrumentation is used for more intricate and dedicated tasks, and does need frequent calibration and careful quality control. As equipment management includes both categories, in the following we use the term “Equipment” to refer to both.

**Table 2:** Examples of general laboratory equipment and laboratory instrumentation

General Laboratory Equipment		Laboratory Instrumentation
Autoclave Automated cover glass/cassettes instrumentation Balance/scale Biological cabinet Centrifuge: • General purpose • Microhematocrit (dedicated, fixed RPM) • Refrigerated • Stand-alone Fume hood Glassware washer Laboratory thermometer Light box Manual pipette Microscope Microtome	Osmometer Oven pH meter Photometers/light-based device Polarimeter Refractometer Rotator Shaker Temperature-controlled Equipment: • Refrigerator • Freezer • Incubator • Water bath • Blood bank transport container Timer Tissue processor Water purifier	Automated tissue stainer Blood cell analyzer Blood chemistry analyzer Blood gas analyzer Blood typing equipment Centrifuge: • Automated cell washing Co-oximeter Densitometer Electrode-based instrument Electrophoresis system Flow cytometer Ion-selective electrode Mass spectrometer Microbial identification instrumentation Nephelometer Pipettor: • Mechanical • Automated Thermal cycler Thin layer chromatograph Urine analyzer

Below, the framework of QMS13-A [21] is used to compare equipment management systems in CAR laboratories with those in the United States (Table 3).

**Table 3:** Policies and implementation of equipment management in laboratory services in the United States and the Central Asian Republics

Equipment Management		Type of laboratories		
		CAR Clinical and Research Laboratories	US and laboratory staff	
			Clinical Laboratories	Research Laboratories
Planning for New Equipment		An annual plan		
Equipment Acquisition		Through MOH^*^	Through the laboratory budget	
Equipment Validation Plan		Not found	Through contracts with manufacturers	
Calibration and Maintenance	General Laboratory Equipment	Annually by NISM	Periodically by manufacturer	Periodically by manufacturer and lab staffs
	Laboratory Instrumentation	Not found	Periodically by manufacturer	Periodically by manufacturer and lab staffs
Quality Control		Not found	Lab staff	
Troubleshooting, Service, and Repair		Technical service according to contract	According to contract by manufacturers	
Decommissioning of Equipment		MOH	Director of laboratory	
Managing Equipment Records		Not found	Equipment master files	

*Ministries of Health in CAR

**National Institutes of Standards and Metrology in CAR

### Planning for acquisition and implementation of new equipment

Clinical and research laboratories in CAR are authorized to provide plans for the need for new equipment (Table 3). These plans, which include technical descriptions and estimates of the cost of requested equipment, must be signed by directors of the government organizations that oversee the laboratories, and then must be sent to the MOH, where the purchase of equipment is centralized. The purchasing of equipment by the MOH can be time consuming and is often dependent on funds provided by donors and development partners, which tend to reflect their particular interests. In some countries of Central Asia, equipment to be purchased must be included in the State System Register. In contrast, the acquisition of new equipment in the USA is entrusted to the individual laboratory or healthcare organization and is carried out through management of the organization’s yearly laboratory capital budget. A common flexible practice in the United States, but not in the CARs, is to enter into leasing agreements with manufacturers rather than purchase equipment (such as chemistry, hematology, blood culture or antimicrobial susceptibility systems). In these agreements, equipment is supplied by manufacturers as long as reagents for these instruments are purchased from the manufacturer. This system offers laboratories flexibility. They do not invest large sums of money in a purchase that “locks them into” a single piece of equipment, and the agreements — which will be for a specified period — typically can include wording that the manufacturer will upgrade or perform periodic calibration of equipment when desired or needed. The absence of this option in the CARs invites the purchase of equipment that will not be maintained and also that will become obsolete.

### Equipment validation plan

A validation plan is essential to ensure that equipment functions as intended in daily work [21]. In the US, CLIA regulations require that validation be performed by the laboratory using the equipment. Initial validation must include an assessment of each test method performed on the equipment for the following parameters: precision (within- and between-run reproducibility); accuracy (bias versus a gold standard measurement); reportable range (the linear range for quantification); and local reference range. If the assay testing procedure requires the use of equipment, any laboratory modification of that equipment must establish the analytical sensitivity and specificity of the modified procedure. The CLSI guidelines include detailed recommendations for these validation studies. In many cases, equipment manufacturers provide technical personnel and procedures to assist laboratories with initial validation, and the laboratories conduct subsequent periodic assessments throughout the life cycle of the equipment according to regulatory requirements and manufacturer specifications. All validation, quality control or maintenance activities must be documented electronically or in manual logbooks. These must be signed by the personnel performing the activity and reviewed by a supervisor or director.

In the CARs, however, an initial validation plan is not used. The laboratory never provides an assessment of accuracy, reportable range and local reference range due to lack of requirements to adhere to international standards such as ISO 15189 and developed regulatory documents. This gap is a remnant of policies implemented during the time of the Soviet Union, where validation of equipment was centralized under the auspices of the Institute of Standardization and Validation because all laboratory equipment was manufactured in USSR. After the collapse of the USSR, laboratories started to use equipment from other countries. Equipment from other countries has different validation requirements and the current NISM does not have certified specialists who can validate and calibrate laboratory equipment manufactured outside of the former USSR. As a consequence, the accuracy of test results in the CARs is not assured as validation plans are uncommon.

### Calibration and maintenance of equipment

Calibration verification, as per ISO, WHO and CLSI guidelines, should be performed according to manufacturer’s recommendations [22]. CLIA regulations require that calibration verification should be performed at a minimum defined frequency (e.g., every 6 months), whenever a complete change of reagents for a procedure is introduced, or when there is major preventive maintenance or replacement of parts of the instrument that may influence test performance [23].

In contrast, calibration verification in CAR laboratories is provided only yearly by National Institute of Standardization and Metrology (NISM) for general laboratory equipment. NISM has concluded that hoods, biologic safety cabinets (BSC) and polymerase chain reaction (PCR) machines are not subject to calibration verification. Laboratory instrumentation is tested annually (without calibration verification) solely to verify that instruments are operational, resulting in receipt of an NISM certificate. In general, the NISM do not have engineers with knowledge for calibration verification for recently purchased equipment such as PCR machines, readers, and cell counters. An NISM certificate is the sole requirement for continued operation in a laboratory, and laboratory directors have determined that calibration verification by distributors of the manufacturer is not necessary. Local distributors for the manufacturer provide free maintenance service only for a limited period, after which service contracts with laboratories need to be renewed. In practice, these contracts are rarely renewed. Technical specifications for some equipment are not available in the Russian language, and thus cannot be understood by laboratory staff. As a consequence, general equipment and laboratory instruments such as hematology analyzers, blood chemistry analyzers, blood typing equipment, flow cytometers, microbial identification instruments, thermal cyclers and BSC are never calibrated after the initial distributor’s service. For example, BSC began to be installed in the CAR clinical laboratories in the 1990’s, but have yet to be tested or certified by NISM [23]. Even if calibration of laboratory instrumentation occurs, records are not kept to document daily, monthly, and quarterly preventative maintenance of equipment in accordance with the manufacturer’s instructions.

In addition, the guidance provided in equipment maintenance documents provided by manufacturers is not part of laboratory practice in Central Asian countries (or most other developing countries). Preventive maintenance is intended to minimize unexpected failure of equipment or instruments so they continue to function as desired. The laboratory needs schedules for preventive maintenance; the manufacturer recommends these schedules. The laboratory needs to follow these schedules [21].

Manufacturers provide instructions for cleaning, adjusting, and replacing disposables on instruments and equipment. At a minimum, preventive maintenance records need to include the following:

- Instrument or equipment identification
- Date and time maintenance is performed
- Maintenance activities performed
- Identity of the person performing maintenance
- Any necessary follow-up actions taken
- Review and approval [21]

Preventive maintenance schedules recommended by manufacturer are in our experience never conducted in CAR laboratories.

### Quality control of examination (analytical) equipment

International laboratory quality standards require a quality control (QC) policy for each instrument or component of the equipment to provide ongoing assurance that performance continues to meet specifications. A documented QC plan is needed for each examination system, which includes installation and maintenance of the equipment, quality of reagents, and skills of the operator to use the equipment. When examination systems have the ability to assay multiple analytes, a QC plan should be established for each analyte or set of analytes. When developing the plan, the laboratory should consider the stability of the equipment, its susceptibility to malfunction or error, and the risk associated with an undetected measurement error or other out-of-specification occurrence. The laboratory should follow the established schedule for frequency and timing of QC, as well as what ranges and types of QC materials should be used. The laboratory should establish limits of acceptability for QC results, and confirm that QC is within the acceptable range before releasing laboratory test results [21]. Laboratories operating according to international standards follow regulatory (CLIA [24]) or accreditation requirements (ISO15189: 2012 [14]) that define the number and frequency of control samples for quantitative assays (often two or three samples above and below the reference range, run daily or on every shift of operation). However, in some CAR laboratories, control materials are used only weekly, which may not be sufficient to ensure consistent, reliable results. Further, and for example, most AIDS Center Laboratories in one CAR country use equipment by one manufacturer, equipment which is supplied with QC program software. However, this software has been switched off by the vendors (personal observation). In addition, most CAR laboratories have not established limits of acceptability for results from their quality control (QC) materials, and have not developed statistical methods to monitor QC performance within individual test runs, or over a series of runs (such as graphical tools like Levey-Jennings plots). Furthermore, many CAR laboratories have not established specific procedures to take corrective action when QC results do not meet criteria for acceptability.

### Troubleshooting, service, and repair

Due to the complexity of modern laboratory equipment, and the sensitivity of test results to subtle changes in equipment performance, laboratories must establish processes to detect and correct instrument malfunction. In the US, most laboratories are required to define their procedures for periodic maintenance, troubleshooting service and repair for all instruments throughout the entire span of their active use. These processes must be performed by qualified personnel (often through contracts with the manufacturers of their equipment). In CARs, troubleshooting, service, and repair are provided by local distributors of the manufacturer’s equipment without charge in the first year after purchase, as a component of the initial purchase price. Some laboratories have long-term contracts with local vendors for maintenance, but solely for broken equipment or replacement of critical parts. These contracts do not include scheduled periodic maintenance and calibration verification of equipment due to the lack of guidelines for equipment management. When the first year of free service ends, a continuing service agreement is generally not purchased due to budgetary constraints. As a consequence, damaged equipment in the CARs commonly remains unrepaired for lengthy periods of time.

### Decommissioning of equipment

According to QMS13-A [21] decommissioning equipment involves a process to ensure the equipment meets the health and safety requirements for the equipment’s next use, such as reassignment to another facility or final disposition to an approved recycling/disposal center. Decommissioning requirements vary according to equipment type and the nature of substances used in operating the equipment. On many occasions, the manufacturer takes responsibility for decommissioning the equipment.

There are no specific requirements for decommissioning equipment in CAR. Every government-sector entity with a laboratory service (for example, AIDS Centers, hospitals and research institutes) has its own team for decommissioning equipment, which is approved by the director of the government entity. The decommissioning team may include a vice director, accountant, head of the laboratory and logistical experts from organizations and laboratories. Depending on equipment status or expected lifespan and typically according to requirements of the Rules for the Decommissioning and Utilization of the Material Values of the State Material Reserve (a Government decree), the team prepares a list of equipment to be decommissioned, which is signed by a director and sent to the MOH for a final decision. The MOH has its own group or department and authority for sign off on the list. Because of these multiple steps, actual decommissioning of problematic equipment is a lengthy process.

### Managing equipment records

Equipment documents and records are an essential part of the quality system. The policies and procedures for installation and then maintenance should be defined in appropriate documents, and keeping good equipment records will allow for thorough evaluation of any problems that arise, as well as necessary inspections by regulatory or accreditation organizations. In the US, laboratories equipment documents and records for both types of equipment (general equipment and laboratory instrumentation) are maintained in equipment master files. In CAR, full equipment documents are ensured for some general equipment such as autoclaves. There are no ‘managing equipment records’ for laboratory instrumentation, aside from the manufacturer’s instructions, for installation and repair and receipt of the annual certificate from the NISM. Thus, accurate auditing of the physical status and performance of equipment in the laboratory is impossible.

## Conclusion

International standards and training to reach those standards have been developed. In CAR laboratories, the approach to quality-assured testing--and in particular the management of laboratory equipment--is not in line with international standards. In the US, management of laboratory equipment depends on direct local administration, compliance with ISO and CLIA regulations, and compliance with federal, state, and local laws. In contrast, management of laboratory equipment is centralized in the CARs, with a number of organizations involved. While the NISMs play an essential role in management of equipment, it is not identified who—NISMs or others —is responsible for the entire range of services needed for standardization and quality management in laboratory services and annual validation of laboratory equipment. An NISM certificate of laboratory instrumentation, both initial and annual, is considered the sole requirement; however, NISMs do not have engineers with the requisite knowledge for calibration verification of laboratory instrumentation. An alternative approach, calibration verification by distributors of the manufacturer, has not been performed consistently. The responsibilities of vendors are not properly defined, and responsible organizations such as the national Ministries of Health are unaware of the current problems in equipment management. Therefore, laboratories often do not ensure essential elements of equipment management such as scheduled calibration, verification and QC. In addition, the recommendations issued by international organizations such as ISO have not been adapted for local conditions, and CLSI and WHO guidelines have not been implemented. These challenges threaten the validity of test results from laboratory services in CAR and serve as a barrier for rapid detection and control of outbreaks of infectious diseases and other public health threats at their source, and therefore represent a threat to global health security.

## Recommendations

The recommendations below provide an approach Ministries of Health may adopt to help ensure accurate test results within CAR laboratories. The recommendations are not exhaustive and focus on equipment. While well-maintained and quality-controlled equipment is only one part of an overall approach to achieving comprehensive quality management in CAR laboratories, it is an essential part.

1. A decision can be made within Ministries of Health that quality assurance of laboratory services must be improved and a national or international quality standard adopted.
2. A Laboratory Quality Unit can be established within the MOH with the mandate to implement, monitor and certify laboratory quality management systems operations according to the national or international standard.
3. Resources to develop a national strategy for the improvement of quality assurance of laboratory services can be provided along with resources to implement it.
4. Resources and training for implementing quality assurance, including quality installation and maintenance of laboratory equipment, can be provided to directors and managers of laboratory services and laboratories.
5. ISO, CLSI and WHO guidelines can be adapted for the conditions of CAR.
6. Regulatory documents can be prepared that clearly describe the responsibilities of each organization with a role in equipment management.
7. Laboratory accreditation programs can be established. These programs can be based on periodic laboratory inspections which ensure that laboratories are completing specific, measurable activities in quality management, similar to laboratory accreditation inspections in developed countries.
8. CARs can define responsibilities of vendors of laboratory equipment. For example, NISMs could establish minimal standards for scheduled maintenance, which can be met for vendors to offer their equipment to CAR laboratories.

## Abbreviations

AIDS: Acquired Immunodeficiency Syndrome
BSC: Biologcal Safety Cabinet
CAR: Central Asia Republics
CDC: Centers for Disease Control and Prevention
CLIA: Clinical Laboratory Improvement Amendments
CLSI: Clinical and Laboratory Standards Institute
ISO: Internatonal Organization for Standartization
LQSI: Laboratory Quality Stepwise Implementation
MOH: Ministry of Health
NISM: National Institute for Standards and Metrology
PCR: Polymerase Chain Reaction
QC: Quality Control
QMS: Qulaity Management System
SLIPTA: Stepwise Laboratory Improvement Process Towards Accreditation
SLMTA: Strenthening Laboratory Management Towards Accreditation
WHO: World Health Organization
